# Management and performance of fattening lambs and goat kids in various rearing systems from Swiss dairy farms

**DOI:** 10.3389/fvets.2025.1644500

**Published:** 2025-08-04

**Authors:** Hanna Voigt, Patrik Zanolari, Nina Maria Keil, Barbara Lutz, Madeleine F. Scriba, Antonia K. Ruckli

**Affiliations:** ^1^Centre for Proper Housing of Ruminants and Pigs, Swiss Federal Food Safety and Veterinary Office, Agroscope Posieux, Posieux, Switzerland; ^2^Clinic for Ruminants, Vetsuisse Faculty, University of Bern, Bern, Switzerland

**Keywords:** gamma globulin, average daily weight gain, health, small ruminant, fattening farm

## Abstract

Many of the lambs and goat kids born annually on dairy sheep and goat farms are not needed for herd replacement and are slaughtered for meat. The goal of this study was to describe rearing and fattening systems for lambs and goat kids from dairy production in Switzerland and to assess their impact on gamma globulin serum levels, health, average daily weight gain (ADG) and mortality. Data from 543 lambs and 247 goat kids from 22 dairy sheep and 17 dairy goat farms in Switzerland was collected. All animals were examined twice (goat kids) or thrice (lambs) in visits V1, V2 and V3 and followed from birth until slaughter. The main rearing systems identified were mother-bound (MB), temporarily mother-bound (TMB) and artificial (ART) rearing. Gamma globulin serum were on average lower in lambs (estimated mean [lower/upper confidence interval]: 1.0 [0.77/1.14] g/dl) than in goat kids (1.3 [1.14/1.56] g/dl; *p* = 0.010) and were higher in younger animals than in older ones (*p* = 0.005). Lambs fed milk feed ad libitum or temporarily ad libitum had higher ADG between V1 and V2 than those fed restrictively (268 [250/285] and 240 [205/274] g/day; *p* = 0.041). Lambs reared TMB had higher ADG between V2 and V3 than those reared MB and ART. Lambs transferred to a fattening farm before weaning had lower ADG between V1 and V2 than lambs remaining on their birth farm (198 [179/217] vs. 255 [243/267] g/day; *p* = 0.003) but higher ADG between V2 and V3 (235 [210/259] vs. 210 [193/229] g/day; *p* = 0.002). Overall mortality was 11.9% in lambs and 6.1% in goat kids. Mortality was higher in lambs that were weaned on fattening farms (18.5%) than in lambs remaining on their farm of origin (10.8%; *p* < 0.001). In conclusion, satisfactory results in health and performance could be achieved in all observed rearing and fattening systems. This highlights the need to examine other factors of the rearing management more closely to conclude on the impact of the various rearing systems on the welfare of these lambs and goat kids.

## 1 Introduction

Sheep and goat milk products remain in consistently high demand in Switzerland, though at lower levels than cow’s milk. In 2023, 21,600 tons of goat’s milk, 6600 tons of sheep’s milk and over 3.7 million tons of cow’s milk were produced (1). Like all mammals, sheep and goats must give birth regularly to initiate milk production. Ewes and goat dams typically have litters larger than one (2) and usually give birth over a short period in spring because of their seasonal reproductive cycle (3), leading to a significant number of births within a short time on dairy farms.

With a replacement rate of under 35% (4, 5), most of the lambs and goat kids born on dairy farms are fattened and slaughtered for meat. However, demand for lamb and goat meat in Switzerland is relatively low (6). Of the 48.43 kg of meat consumed per person in Switzerland in 2023, only a small portion derived from small ruminants (1.01 and 0.06 kg of lamb and goat meat, respectively). Furthermore, dairy sheep and goat breeds are selected primarily for a high milk yield and quality, making selection for average daily weight gain (ADG) and meat traits of their purebred offspring not a main priority (7, 8). This situation makes the fattening of these surplus animals economically unattractive while their rearing demands significant time and labor from dairy farmers.

However, good management and husbandry practices during rearing and fattening are essential for the welfare of lambs and goat kids as they commonly face health problems such as diarrhea, respiratory disease and infections of the umbilicus or joints during early life (9). Death often occurs in these young animals within the first days of life with half of pre-weaning deaths occurring on the day of birth (10, 11). Mortality rates in the first five to seven days of life range from 10 to 25% in lambs and from 7 to 51% in goat kids (12) and vary greatly across farms (13, 14). Adequate colostrum intake, in both quantity and quality, is essential for transferring immunity to lambs and goat kids (15) as it may be associated with better health outcomes and lower mortality (16, 17). However, not all studies confirm this causality (18), and low levels have also been observed in surviving lambs (19, 20). Variation in rearing systems and further farm management practices may influence both the concentration of gamma globulin in colostrum and the ability of lambs and goat kids to absorb ingested gamma globulin (21, 22) and thus also the animals’ health. Morbid lambs and goat kids have been shown to have a lower ADG (23). Thus, ADG should be an indicator of health, which is essential for good welfare and additionally relevant for the economic situation for the farmers.

Little is known about the rearing and fattening of surplus dairy lambs and goat kids in Switzerland. Studies conducted in other countries observed that lambs or goat kids are either left with their dams throughout the entirety of the suckling period, are allowed to suckle their dams periodically at certain times of day or are separated early and raised artificially (7, 24–26). If raised artificially, feeding methods, milk feed types and milk feeding schedules vary widely (27, 28). Some animals are relocated to fattening farms (7, 24), which may further impact their development. Although studies on different rearing systems (such as mother-bound vs. artificial) exist, most focus on meat breeds rather than dairy breeds (29–31). It is reasonable to assume that the rearing systems influence both the health and growth performance of dairy lambs and goat kids. Understanding these dynamics is crucial for developing management practices that improve the welfare of these animals.

To address this need, we conducted a comprehensive, longitudinal study of lambs and goat kids from various Swiss dairy farms, combining on-farm observation with data from the national animal identification database, tracking them from birth until max. 12 months of age. The objectives of this study were (1) to describe the rearing and fattening systems used for lambs and goat kids not intended for breeding on Swiss dairy sheep and goat farms, (2) to assess their gamma globulin status, health, ADG and mortality, and (3) to identify management factors of rearing and fattening that promote health and daily weight gains in lambs and goat kids.

## 2 Materials and methods

### 2.1 Farms and data collection

**Figure 1:**
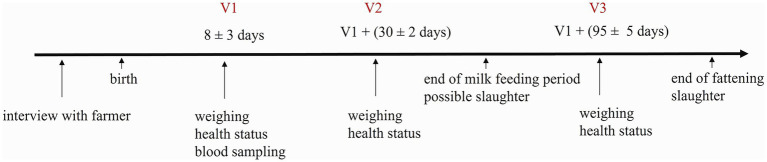
Timeline of the experimental schedule of the three visits (V1, V2, V3) conducted on the sheep and goat farms. Goat farms were visited only twice (V1, V2).

From November 2021 to June 2023, data was collected on 23 dairy sheep and 20 dairy goat farms in Switzerland. Sheep farms were visited three times whereas goat farms were visited two times. The first visit (V1) took place in the first 3 weeks after birth, the second visit (V2) 30 ± 2 (mean ± standard deviation) days after V1 and the third visit (V3; sheep farms only) 95 ± 5 days after V1 (Figure 1). Animals were furthermore tracked via the official Swiss national animal identification, registration and movement database (Animal Tracing Database; ATD) (32), until the age of 12 months for assessment of animal mortality and slaughter ages (see Section 2.3). All farmers were recruited by phone or letter. Participation was voluntary, and written consent was given by all participating farmers. Inclusion criteria were active dairy production and lambing times between September and April.

If the animals were sold to a fattening farm between V1 and V3, the subsequent data collection was conducted on the respective fattening farm. Fattening farms were categorized as farms to which lambs or goat kids were transported with the objective of fattening. The transfer had to be registered in the ATD. One dairy sheep farm, three dairy goat farms and one goat kid fattening farm had to be excluded from the study owing to insufficient data regarding animal-based indicators. This exclusion resulted in data from 22 dairy sheep farms, 17 dairy goat farms, eight lamb fattening farms and one goat kid fattening farm. The animal study was reviewed and approved by the Committee of Animal Experiments of the Canton of Zurich (Ethics Reference No: TG08_2021) and was in accordance with the Swiss guidelines for animal welfare.

### 2.2 Farm characteristics and management

An interview with the farmer was conducted either before or during V1, usually on site by the author HV. The interview included questions on general farm data (e.g., herd size, breeds, affiliation to a label; see Table 1) and data on birth management, colostrum phase, the rearing system, suckling systems, weaning age, fattening system and usual age at slaughter. If the answers contained ranges of values such as a range in milk feed allowance per animal, the average value was calculated. If any management changes relevant to the sampled animals were reported by the farmer during the subsequent farm visits, the information was adjusted accordingly.

**Table 1:** Farm characteristics of the 22 dairy sheep and 17 dairy goat farms and their associated fattening farms.

Farm details	Sheep farms	Goat farms
Dairy farms (*n*)	22	17
Lactating dams (*n*) (median) (range)	125 (25–600)	60 (18–120)
Organic production (%)	50	47
Farming as main source of income (%)	96	77
Associated with herd book (%)	64	71
Use of fattening breeds for mating (%)	64	41
Selling of lambs or goat kids to fattening farms before weaning (%)	32	6
Selling of lambs or goat kids to fattening farms after weaning (%)	23	0
Fattening farms (*n*)	8	1
Animals fattened per farm and year (*n*) (median) (range)	500 (25–3,000)	1,500
Organic production (%)	38	0
Farming as main source of income (%)	75	100
Buying of unweaned lambs and/or goat kids (%)	38	100

### 2.3 Animal-based data collection

On each dairy farm, 10% of the annually expected offspring (with a minimum number of 10 and a maximum number of 34 lambs or goat kids per farm) were selected at V1 for the animal-based data collection. On two sheep farms, two distinct sample groups were chosen to reflect two different management practices and thus more than 10% of the expected offspring were selected. Animals intended for herd replacement were excluded from the sample when this information was available. This selection resulted in an initial sample of 543 lambs and 247 goat kids. The number of animals declined across the subsequent visits (V2 and V3) owing to factors such as death, slaughter or missing data. The sample sizes analyzed were 539 lambs and 235 goat kids at V1, 476 lambs and 212 goat kids at V2, and 379 lambs at V3. No goat kids were assessed at V3 because most had been slaughtered by that time. The numbers can also be found in a table format in the Supplementary Table S1.

#### 2.3.1 Health status and average daily weight gain

The health status of the selected lambs and goat kids (10% of the annually expected offspring) was examined by a trained veterinarian (author HV or BL) at each visit for six clinical indicators by using a standardized method (Table 2). Each indicator was scored as “present” or “absent.” If no note was made on a specific indicator, absence of clinical signs was assumed. For statistical analysis, the results of the health indicators were summarized into one health score: 0 (healthy, no deviation from the healthy norm, all indicators scored as “absent”) or 1 (morbid; at least one of the clinical indicators scored as “present”). The indicator “general condition” was excluded from this health score due to different handling procedures of the animals before the assessment, which influenced their stress level and thus the interpretation of the results. The lambs and goat kids were individually weighed at each visit by using a transportable scale. The ADG (g/day) was calculated for the periods between V1 and V2, and for lambs also between V2 and V3 and between V1 and V3.

**Table 2:** Definition of the six health indicators for assessing on-farm welfare examined for the individual lambs and goat kids and used to calculate the overall health score.

Health indicator	Definition
General condition	Signs of disturbed health (hunched posture, drooping ears, introversion)
Lameness, joint swelling	Obvious movement disorders (lameness, unnatural posture or movement) and/or enlarged joints, dolent on palpation and/or with an increase in synovial fluid
Skin lesions	Orf-like lesions apparent on visual inspection of the (muco)cutaneous areas of the head
Respiratory quality	Distinct accentuation and/or by-noise in lung auscultation and/or silent lung in combination with other findings
Umbilical disorders	Clinical symptoms of umbilical disorders such as swelling and discharge
Fecal soiling, diarrhea	Observed diarrhea and/or at least medium-grade scours (soiling in the anal area that firmly extends to wool in lambs or covers most of the perineal area in goat kids)

#### 2.3.2 Blood sampling and gamma globulin measurement

At V1, blood samples (4.5 mL) were collected from five to eight out of the selected lambs and goat kids per farm via jugular vein puncture. Each animal was sampled only once. The serum was centrifugated and then frozen until shipment to IDEXX Westkornheim, Germany, where gamma globulin content was measured using agarose serum electrophoresis (33). Total protein concentrations were determined using the biuret method. The gamma-fraction was determined, and absolute concentration was automatically calculated based on the total serum protein level. In total, blood samples were collected from 137 lambs and 95 goat kids. However, owing to hemolysis and missing data, 6 lambs and 9 goat kids had to be excluded from the analysis and the sera of 131 lambs and 86 goat kids were finally included in the statistical analysis.

#### 2.3.3 Slaughter age and mortality

Individual animals’ slaughter ages as well as mortality rates for the initially selected animals (10% of the annually expected offspring) were calculated with the information provided in the ATD up to 12 months of age. Overall mortality rates were calculated for lambs and goat kids separately until 365 days of age. Furthermore, mortality was assessed regarding the place of fattening. The data basis for this analysis was the full initial sample. Animals that were not correctly registered in the ATD or to which we could not attribute an ear tag number were excluded from the analysis of mortality, leaving 521 lambs and 229 goat kids. Additionally, for the comparative analysis regarding place of fattening, all animals that were given to another farm that we did not visit and therefore did not confirm whether they were dairy farms and bought animals for restocking or bought the animals with the objective of fattening were excluded, leaving a sample size of 508 lambs. For slaughter ages, all available slaughtered animals that were slaughtered until 365 days of age were included (lambs: *n* = 396, goat kids: *n* = 194).

### 2.4 Statistical analyses

Statistical analysis and data visualization were conducted using R [Version 4.4.1; R Core Team, 2024 (34)]. Descriptive results of the animal-based indicators are provided in the Supplementary Table S2.

Fixed effects were defined and categorized based on the interview and individually assigned to reflect the individual animals’ experiences as follows: For “contact dam V1,” it was categorized if a lamb or goat kid was either “still with its dam” or “without its dam” at V1.

“Rearing system” was categorized based on the duration lambs or goat kids remained with their dams during the whole rearing period. If a lamb or goat kid was separated from the dam after the time allotted for colostrum ingestion (colostrum phase), it was assigned to “artificial” (ART) rearing. If a lamb or goat kid stayed with its dam until weaning, it was assigned to “mother-bound” (MB) rearing. In cases where the young initially stayed with its dam but was later separated during the suckling period for artificial rearing, it was assigned to “temporarily mother-bound” (TMB) rearing.

Because changes in milk feed allowance were common throughout the suckling period, the data collected from the interview was divided into two phases for analysis: the “beginning of the suckling period” (immediately after the colostrum phase) and the “end of the suckling period” (just before weaning). Suckling period refers to the full time that lambs were offered milk feed, regardless of whether they still were with their dam or not.

The two fixed effects “milk feed allowance beginning of suckling period” and “milk feed allowance end of suckling period” were defined based on the following criteria: the milk feed allowance was defined as “ad libitum” if milk feed was always available without restrictions on quantity or if it was provided via an automated system that lacked individual animal recognition and specific feeding times or if lambs and goat kids were suckled by their dams. Milk feed allowance was defined “temporarily ad libitum” if there were no restrictions on the quantity consumed per feeding but temporary limitations on access to milk throughout the day. This restriction was achieved either by removal of the artificial feeding device after all young stopped showing immediate interest or by separation of dams and young at night (as practiced by one dairy sheep and two dairy goat farms). If restrictions were imposed on both the frequency of access to milk feed and the amount offered, the milk feed allowance was defined as “restrictive.”

The fixed effect “weaned from milk V2” described if an animal was already weaned from milk (“yes” or “no”) at V2. “Place of weaning” described whether an animal was weaned on the birth farm or if weaning took place after transfer to a fattening farm. “Place of fattening” described whether a lamb or goat kid either remained on its birth farm for the full duration of fattening or if it was transferred and fattened on a fattening farm. The fixed effect “concentrate feed” categorized whether lambs and goat kids were offered any concentrate (“yes” or “no”) based on the definition of Suisse Bilanz (35) after V2.

As outcome variables, gamma globulin status (in g/dl at V1), health scores (healthy/morbid at V1, V2, V3), ADG (in g/day in the periods V1–V2 and V2–V3) and mortality in lambs (died/did not die) were analyzed with mixed models with “farm” as random effect. The model for gamma globulin status and health V1 combined both species. For all other models, the models were set up species specific because the management of lambs and goat kids differed substantially between species after V1. No models were calculated for goat kids for health at V3 or ADG V2–V3 because most goat kids had been slaughtered after V2. Data from one dairy goat farm (*n* = 12 goat kids) were excluded from the statistical analysis for health V2 and ADG V1–V2 owing to its unique management practices regarding the rearing compared with the other farms.

The mixed models analyzing gamma globulin status and ADG were calculated using the “lmer” function of the “lme4” package (36), whereas models for the health score were run using the “glmer” function of the “glme” package (37). Model assumptions were checked visually through graphical analysis of residuals (normality and homoscedasticidy). Each global model was tested against its null model to assess the overall explanatory value of the models (with *p* ≤ 0.05 as level of significance). If this global test was significant, dummy variables with sum contrasts were used for the fixed effects included in the models to evaluate the individual factor’s statistical influence on the outcome variable. The *p*-values were obtained by comparing the full model with models reduced by single factors, using the “pbkrtest” package (38). Model estimates and confidence intervals [upper/lower] were calculated with the “effect” package (39). The models that were run were:


$$\begin{array}{l} \text{Gamma globulin}\sim \mu +\text{Species}+\text{Contact}\;\mathrm{dam}\;\mathrm{V1}+\\ \text{Health}\;\mathrm{V1}+\mathrm{Age}+(1|\text{FarmID}) \end{array}$$

HealthV1~μ+Rearing system+Species+(1∣FarmID)

$$\begin{array}{l} \text{Health}\;\mathrm{V2}\;\text{lambs}\sim \mu +\text{Rearing system}+\text{Milk feed allowance}\;\\ \mathrm{end}\;\text{of suckling period}+\text{Place of weaning}+(1|\text{FarmID}) \end{array}$$

HealthV2goat kids~μ+Rearing system+(1∣FarmID)

$$\begin{array}{l} \text{Health}\;\mathrm{V3}\;\text{lambs}\sim \mu +\text{Rearing system}+\text{Milk feed allowance}\;\\ \mathrm{end}\;\text{of suckling period}+\text{Place of fattening}\;(1|\text{FarmID}) \end{array}$$

$$\begin{array}{l} \mathrm{ADG}\;V1–\mathrm{V2}\;\text{lambs}\sim \mu +\text{Rearing system}+\text{Milk feed allowance}\;\\ \text{beginning of suckling period}+\text{Milk feed allowance}\;\mathrm{end}\;\text{of}\;\\ \text{suckling period}+\text{Place of weaning}+\text{Weaned from}\;\\ \text{milk}\;\mathrm{V2}+\text{Health}\;\mathrm{V1}+\text{Health}\;\mathrm{V2}+(1|\text{FarmID}) \end{array}$$

$$\begin{array}{l} \mathrm{ADG}\;V1–\mathrm{V2}\;\text{goat kids}\sim \mu +\text{Rearing system}+\text{Milk feed}\;\\ \text{allowance beginning of suckling period}+\text{Milk feed}\;\\ \text{allowance}\;\mathrm{end}\;\text{of suckling period}+\text{Health}\;\mathrm{V1}+\\ \text{Health}\;\mathrm{V2}+(1|\text{FarmID}) \end{array}$$

$$\begin{array}{l} \mathrm{ADG}\;V2–\mathrm{V3}\;\text{lambs}\sim \mu +\text{Rearing system}+\text{Milk feed allowance}\;\\ \mathrm{end}\;\text{of suckling period}+\text{Place of fattening}+\text{Health}\;\mathrm{V3}+\\ \text{Concentrate feed}+(1|\text{FarmID}) \end{array}$$

Mortality lambs~μ+Place of fattening+(1∣FarmID)

## 3 Results

### 3.1 Farm characteristics

#### 3.1.1 Dairy farms

The farms visited varied considerably regarding their farm characteristics (Table 1). Farms were located in 15 of the 26 regions of Switzerland with 36% of dairy sheep farms and 65% of dairy goat farms being in the mountainous region. Herd sizes varied greatly across farms. The distribution between organic and conventional farms was about 50% in both species. The main dairy sheep breeds were Lacaune and East Friesian dairy sheep. The dairy goat farms had a wide variation of Swiss goat breeds, mainly Alpine goat, Saanen goat, Peacock goat and Grison Striped goat. 14 dairy sheep and 7 dairy goat farms used fattening breeds to sire lambs and goat kids with the aim to improve the meat performance with crossbred offspring. For this, exclusively Boer breed sires were used in goats. In sheep, a greater variety of sire breeds was prevalent, predominantly Texel but also Suffolk, Beltex, Charollais, Berrichon du Cher and some others.

#### 3.1.2 Fattening farms

Of the nine fattening farms participating in the study, five exclusively fattened sheep whereas four fattened both species (Table 1). Some fattening farms bought their fattening stock still relying on milk feed whereas others only bought animals that had already been weaned on their dairy farm. None of the fattening farms that bought unweaned lambs or goat kids were organically managed whereas three of the five fattening farms exclusively buying weaned lambs were organic. Fattening farms bought their fattening stock from one dairy farm to up to 20 dairy farms. On some farms, animals were bought from animal traders and animal markets so that the number of farms of origin could vary.

### 3.2 Management of rearing

#### 3.2.1 Birth and colostrum management

Lambing mostly took place during winter (December to February) and early spring (March to April), although sheep farms occasionally started their lambing season in fall (September to November). Most sheep and goat dairy farmers (91% of sheep farms and 88% of goat farms) allowed their lambs or goat kids to suckle their dams for up to 5 days postpartum for ingestion of colostrum. To ensure an undisturbed colostrum intake, 11 sheep farms and 9 goat farms generally separated the dam and her offspring for at least 12 h after birth whenever possible.

#### 3.2.2 Rearing and fattening

The average age of the lambs and goat kids at V1 was 8 ± 3 days in both species. Most farms (11 dairy sheep and 11 dairy goat farms) reared the lambs and goat kids mainly in an ART system, while five dairy sheep and five dairy goat farms reared their lambs and goat kids mainly in an MB system. Six dairy sheep farms and one dairy goat farm had a TMB system (Table 3). Some dairy farms used the different rearing systems either simultaneously or alternatingly depending on the specific situation (such as milk contingents by dairies or fluctuating demands due to the season). If the animals were not given to fattening farms, fattening animals were usually reared in the same way as those kept for herd replacement.

**Table 3:** Distribution of the three main rearing systems (MB, mother-bound; TMB, temporarily mother-bound; ART, artificial) used on the dairy and fattening farms raising lambs or goat kids and their methods of milk feeding at the beginning and end of the suckling period.

Rearing system	Lambs			Goat kids		
	MB	TMB	ART	MB	TMB	ART
Dairy farms (*n*)	5	6	11	5	1	11
*Milk feeding at beginning of suckling period*						
Feeding by (*n* farms)						
Dam	5	6	–	5	1	–
Teat bucket	–	–	8	–	–	6
Automatic feeders	–	–	3	–	–	4
Trough	–	–	–	–	–	1
*Milk feeding at end of suckling period*						
Dairy farms (*n*)	5	3	8	5	1	10
Fattening farms (*n*)	–	2	2	–	0	1
Feeding by (*n* farms)						
Dam	5	–	–	–	–	–
Teat bucket	–	2	9	–	1	–
Automatic feeders	–	3	1	–	–	10
Trough	–	–	–	–	–	1

When lambs and goat kids were kept with their dams (MB and TMB), they were kept either in the full herd or in smaller subgroups. Sizes of subgroups could vary greatly between two or three dams with their young to up to 45 dams with their young. For TMB and ART rearing systems, teat buckets and automatic feeding systems were the main artificial feeding systems. Most lambs were fed with cow milk or milk mixtures, whereas the majority of goat kids was fed with cow milk replacer. The amount and frequency of milk feed offered varied between farms (Table 4). Toward the end of their suckling period, 212 lambs were fed ad libitum, 53 temporarily ad libitum and 211 were fed restrictively, whereas goat kids were mostly fed ad libitum (166 goat kids) or temporarily ad libitum (34 goat kids).

**Table 4:** Milk allowance and feeding frequency on sheep and goat dairy and fattening farms that provided milk feed “restrictively” (restrictions were imposed on both the frequency of access to milk feed and the amount offered).

Milk feeding system	Lambs	Goat kids
Milk feeding system at beginning of suckling period		
Farms (dairy) feeding restrictively (*n*)	5	5
Milk allowance per animal per day (l/day) (median) (range)	1.1 (0.8–1.6)	1.5 (1.0–2.4)
Milk feeding bouts allowed per day (*n*) (median) (range)	3 (2–6)	2 (2–3)
Milk feeding system at end of suckling period		
Farms (dairy and fattening) feeding restrictively (*n*)	8	1
Milk allowance per animal per day (l/day) (median) (range)	1.3 (0.6–1.6)	2.3 (–)
Milk feeding bouts per day (*n*) (range)	2.5 (2–8)	2 (–)

The duration of milk feeding ranged from 4 to 10 weeks for lambs and 5 to 16 weeks for goat kids. By Swiss legislation, unlimited access to rough feed like hay has to be granted to lambs after 2 weeks of age (40). Weaning was mostly abrupt in lambs. Only six of the sample groups were weaned gradually (by reduction in the amount of milk feed offered or in the frequency of milk feeding bouts). Goat kids were usually slaughtered before weaning, with weight, age and the Easter season being the most important factors determining the time of slaughter. The goat kids of a single dairy farm were sold to finishing farms with an average age of 8 ± 3 days (mean ± standard deviation).

**Figure 2:**
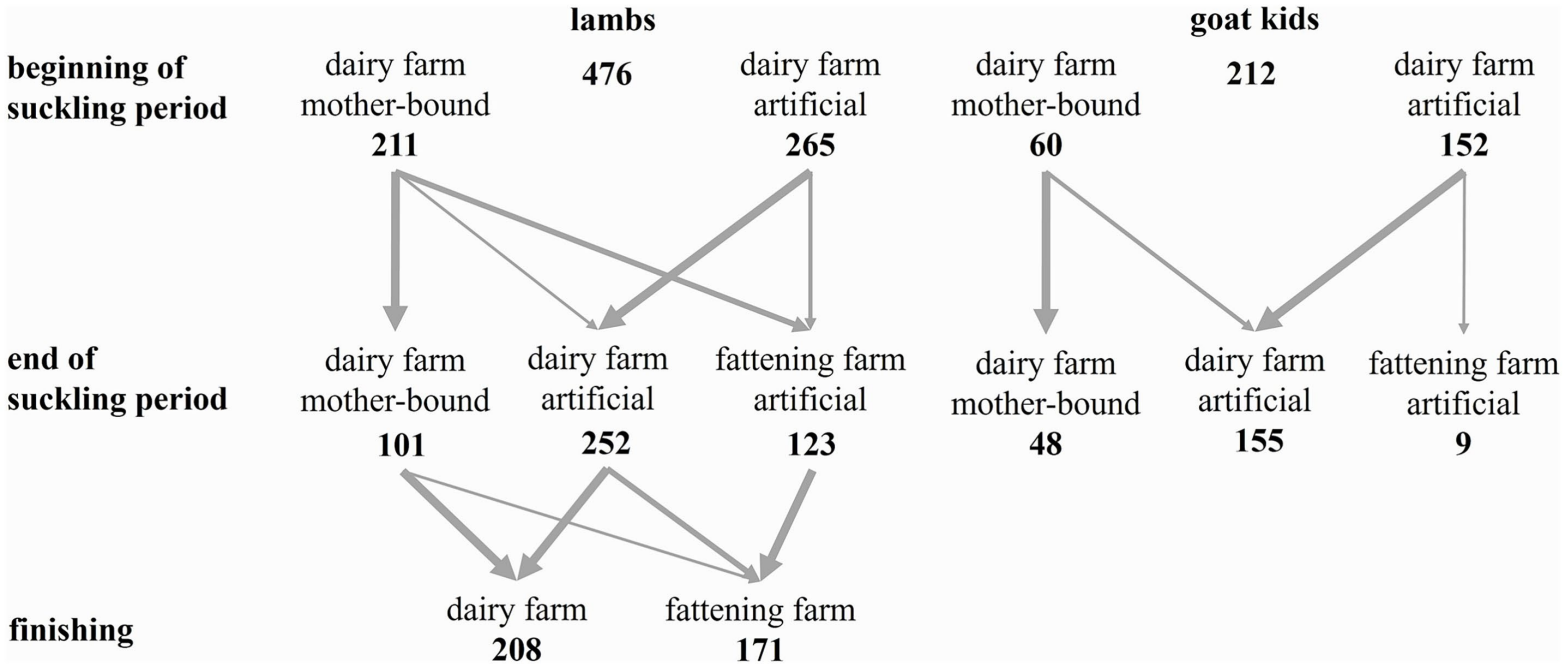
Number of lambs and goat kids in relation to the rearing systems applied at three stages of rearing (beginning and end of suckling period and finishing) and place of fattening (*n*). At finishing, *n* is reduced to 379 lambs because a number of lambs could not be included owing to missing data (e.g., owing to slaughter or death).

Lambs were either slaughtered as suckling lambs or fattened to a live weight of around 40 to 50 kg. Of the 476 lambs and 212 goat kids with complete animal data (e.g., management data, health data, ADG data), 123 lambs were sold to fattening farms before weaning (25.8%, Figure 2) at an average age of 13 ± 4 days. Of the 379 lambs remaining at V3, 54.9% were fattened on their birth farms. The remaining lambs were finished on fattening farms with 57.3% of them having previously been weaned on their birth farms. In these, age at transferal averaged 63 ± 23 days. Several of the dairy farms visited gave their lambs to the same fattening farms. The solid feed that was offered after weaning consisted usually of grass, hay or silage, whole plant corn pellets and in the case of 210 lambs concentrate feed by the definition of Suisse Bilanz (35).

#### 3.2.3 Slaughter age and mortality

Lambs were slaughtered at an average age of 188 ± 74 days (*n* = 396) and goat kids at an average age of 67 ± 32 days (*n* = 194). The overall mortality calculated from the ATD was 11.9% in lambs and 6.1% in goat kids. Lambs that were transported to fattening farms before weaning had a higher mortality rate (18.5%) than lambs fattened on their dairy farms (10.8%) and lambs that were transferred to a fattening farm after weaning (5.4%; *p* < 0.001).

#### 3.2.4 Marketing

Lambs were either sold directly to slaughterhouses or local butcheries or indirectly via animal traders or animal markets. Direct marketing to private consumers was occasionally done on some of the farms. Similarly, the lambs of four fattening farms were mainly sold via animal traders, whereas four fattening farms gave their lambs and goat kids to a local butcher. Of the goat farms, the majority used direct marketing with about half of farms selling some or all to butcheries or bulk buyers.

### 3.3 Gamma globulin serum levels

**Figure 3:**
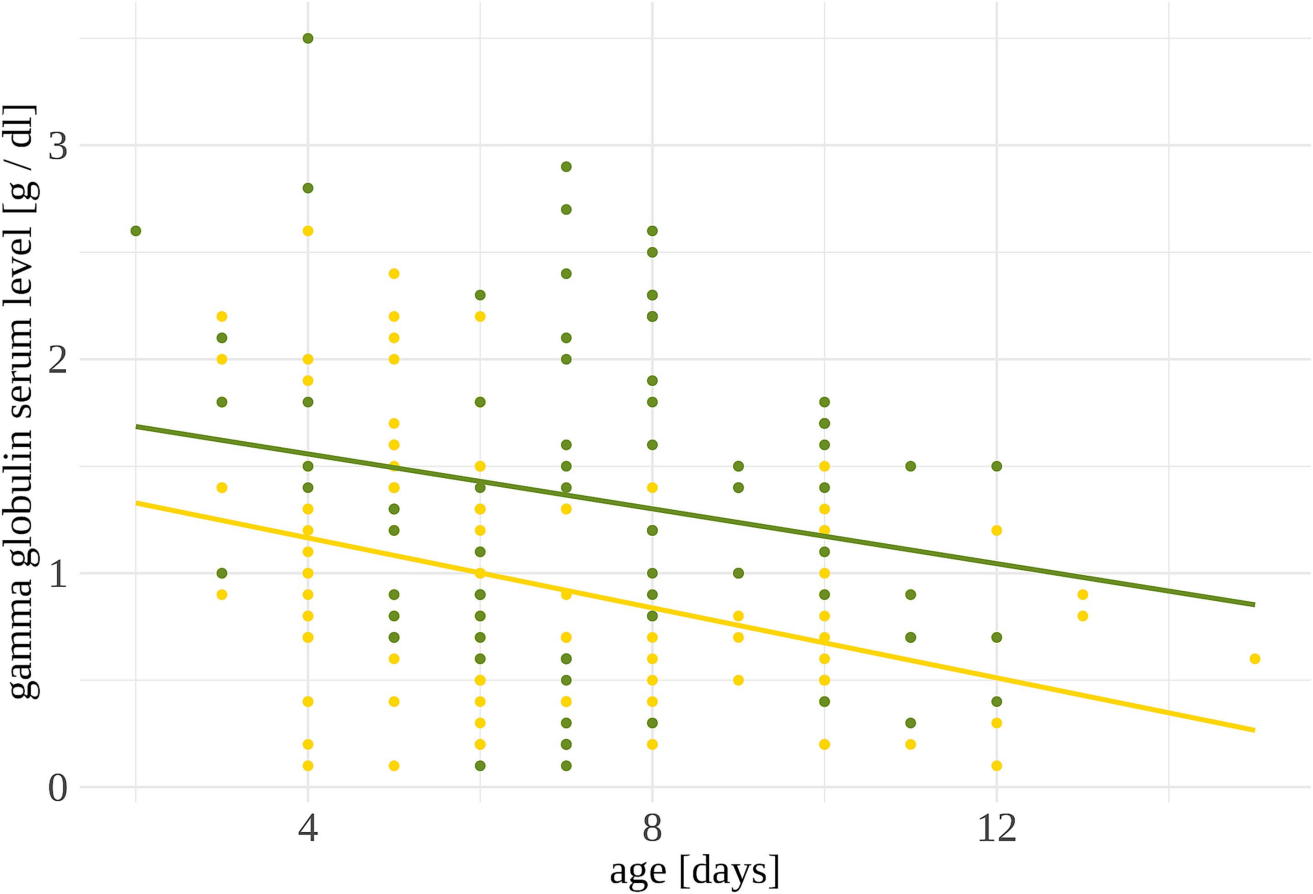
Gamma globulin serum levels in blood samples of lambs (yellow, *n* = 131) and goat kids (green, *n* = 86). Each dot represents the gamma globulin serum level of one animal. Animals were only tested once.

On average, gamma globulin serum levels were lower in lambs (estimated mean [lower/ upper confidence interval]: 1.0 [0.77, 1.14] g/dl) than in goat kids (1.3 [1.14, 1.56] g/dl; *p* = 0.010; Table 5). The values of the samples ranged from 0.1 to 2.6 g/dL in lambs and from 0.1 to 3.5 g/dL in goat kids. The gamma globulin serum levels were higher in younger lambs and goat kids than in older ones (*p* = 0.005; Figure 3). Neither the health score at V1 nor the rearing system at V1 showed statistical influence on the gamma globulin serum levels.

**Table 5:** Fixed effects included in the different mixed models and their *p*-values (boldface indicates statistical significance), with farm included as random effect (ADG = average daily weight gain; V1, V2, V3 = visit 1, 2, 3).

Outcome variable	Fixed effect	*p*-value
Gamma globulin (g/dl) 131 lambs 96 goat kids	Global model	**0.009**
	Species	**0.010**
	Contact dam V1	0.791
	Health V1	0.819
	Age	**0.005**
Health V1 (categorical) 539 lambs 235 goat kids	Global model	0.342
	Rearing system	
	Species	
Health V2 lambs (categorical) 476 lambs	Global model	0.313
	Rearing system	
	Milk feed allowance end of suckling period	
	Place of weaning	
Health V2 goat kids (categorical) 200 goat kids	Global model	**0.004**
	Rearing system	**0.004**
Health V3 lambs (categorical) 379 lambs	Global model	0.383
	Rearing system	
	Milk feed allowance end of suckling period	
	Place of fattening	
ADG V1–V2 lambs (g/day) 467 lambs	Global model	**<0.001**
	Rearing system	0.159
	Milk feed allowance beginning of suckling period	0.330
	Milk feed allowance end of suckling period	**0.041**
	Place of weaning	**0.003**
	Weaned from milk V2	0.910
	Health V1	0.430
	Health V2	**<0.001**
ADG V1–V2 goat kids (g/day) 200 goat kids	Global model	0.135
	Rearing system	
	Milk feed allowance beginning of suckling period	
	Milk feed allowance end of suckling period	
	Health V1	
	Health V2	
ADG V2–V3 lambs (g/day) 379 lambs	Global model	**<0.001**
	Rearing system	**0.002**
	Milk feed allowance end of suckling period	0.590
	Place of fattening	**0.002**
	Health V3	0.930
	Concentrate feed	0.668
Mortality lambs (categorical) 521 lambs	Global model	**<0.001**
	Place of fattening	**<0.001**

### 3.4 Health indicators

At V1, 17.6% of lambs and 11.5% of goat kids showed signs of at least one health issue (Table 6), with umbilical disorders being most prevalent in lambs and fecal soiling or diarrhea in goat kids. At V2, 30.5% of lambs and 16.5% of goat kids were affected with the main issues being fecal soiling or diarrhea and orf-like skin lesions in lambs and orf-like skin lesions in goat kids. At V3, 22.2% of the lambs had health findings, of which respiratory quality was most predominant. Morbidity varied greatly across farms in both species. In lambs, farm morbidity ranged from 0 to 43.3% at V1, 0–83.3% at V2 and 0–53.3% at V3. In goat kids, farm morbidity ranged from 0 to 75.0% at V1 and from 0 to 100% at V2. At V2, MB goat kids had a better health status than ART goat kids (*p* = 0.004; Table 5). No other statistical effects of the models’ fixed factors were found on the health status at V1, V2 and V3 in lambs or on the health status at V1 in goat kids (Table 5).

**Table 6:** Prevalence of health indicators in all lambs and goat kids assessed at the three visits on dairy (V1, V2, V3) or fattening farms (V2, V3) as defined in Table 2.

Indicator	Lambs			Goat kids	
Visit	V1	V2	V3	V1	V2
Number of farms (*n*)	22	19	19	17	16
Number of animals (*n*)	539	476	379	235	200
General condition (%)	5.0	5.3	3.7	2.6	4.0
Lameness, joint swelling (%)	1.9	2.5	4.0	1.3	0.5
Skin lesions (%)	2.0	10.7	0.0	0.0	9.0
Respiratory quality (%)	3.5	7.5	14.0	0.4	4.0
Umbilical disorders (%)	7.8	0.6	0.0	3.0	1.5
Fecal soiling, diarrhea (%)	3.9	12.8	5.3	6.8	2.0

“General condition” was excluded for assessing the overall health score.

### 3.5 Average daily weight gain

**Figure 4:**
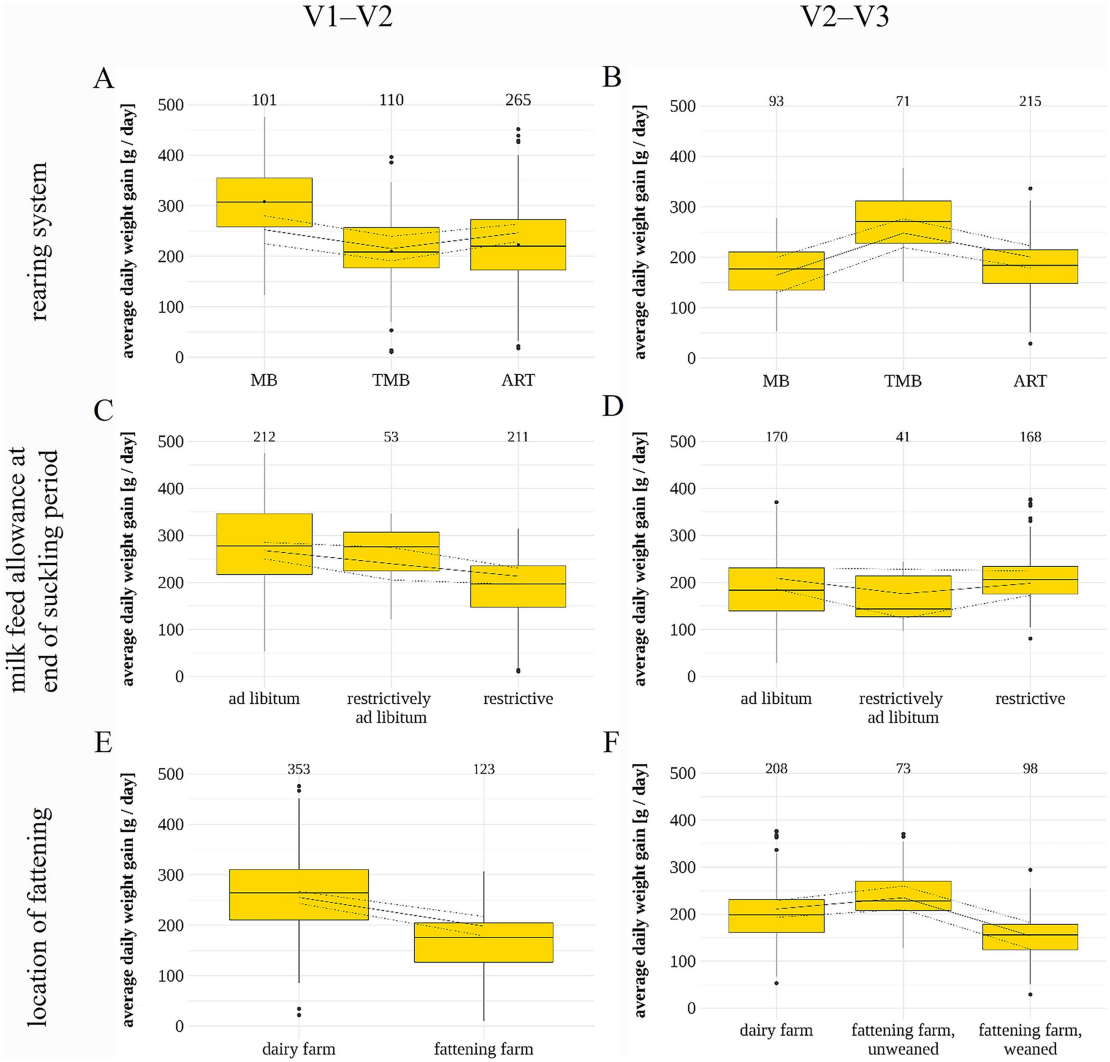
Average daily weight gain of lambs during the first phase of rearing (V1–V2) and the second phase of rearing (V2–V3) in relation to the three rearing systems (MB, mother-bound; TMB, temporarily mother-bound; ART, artificial) **(A,B)**, milk feed allowance at the end of the suckling period **(C,D)** and the location of fattening **(E,F)**. Box plots of the raw data with number of animals included (above) and model estimates (grey line) with confidence intervals (grey dotted lines).

The ADG of the lambs was 238 ± 86 g/day (range: 10 to 476 g/day) in the first phase of rearing (V1–V2) and 196 ± 63 g/day (range: 29 to 377 g/day) in the second phase of rearing (V2–V3). During the first phase of rearing, lambs that were fed milk feed ad libitum or temporarily ad libitum at the end of the suckling period had a higher ADG (268 [250/285] g/day and 240 [205/274] g/day) than restrictively fed lambs (213 [196/230] g/day; *p* = 0.041; Table 5; Figure 4). Lambs that were scored as healthy at V2 had a higher ADG than morbid ones (250 [239/261] vs. 218 [205/232] g/day; *p* < 0.001). Furthermore, lambs weaned on the dairy farms had a higher ADG (255 [243/267] g/day) than those weaned on fattening farms (198 [179/217] g/day; *p* = 0.003). No effect was found for the rearing system or the milk feed allowance at the start of the suckling period. During the second phase of rearing (V2–V3), TMB lambs had a higher ADG (248 [219/277] g/day) than ART (201 [179/223] g/day) and MB lambs (164 [130/199] g/day; *p* = 0.002; Figure 4). Lambs that were sold before weaning and then weaned on the fattening farm had a higher ADG during V2–V3 than those fattened on dairy farms or sold after weaning (235 [210/259], 211 [193/229], 154 [125/182] g/day, respectively; *p* = 0.002). No effect was found for the milk feed allowance at the end of the suckling period, the health at V3 and the provision of concentrate feed (Table 5). Over the entire rearing period (V1–V3), ad libitum-fed lambs had an on average higher ADG of 223 ± 50 g/day than lambs fed milk restrictively (207 ± 44 g/day) or temporarily ad libitum (193 ± 39 g/day). For the rearing system, TMB lambs had the comparatively highest ADG (216 ± 53 g day), followed by MB (216 ± 47 g/day) and ART (198 ± 36 g/day).

The ADG V1–V2 of the goat kids was 227 ± 62 g/day (range: 39 to 386 g/day). No difference was found in the ADG V1–V2 regarding the rearing system, the milk feed allowance at the beginning of the suckling period, the milk feed allowance at the end of the suckling period and the health status at V1 and V2 (Table 5).

## 4 Discussion

In this exploratory study, we assessed a sample of lambs and goat kids born on Swiss dairy farms during their first months of life through visits and further through the systematic tracking of the respective animals in the ATD. We described their rearing and fattening systems and assessed their gamma globulin serum level, health status and ADG during three farm visits.

Although a considerable number of animals were included in this study, please note that the 22 dairy sheep and 17 dairy goat farms and the associated nine fattening farms are only a selection and may not be representative of all farms in Switzerland. Furthermore, the voluntary nature of the study possibly caused a bias of farms with a well-working management because participation of very unsuccessful farms seems unlikely. Nevertheless, with participating farms from the majority of Swiss regions, we were able to showcase a wide variety of dairy and fattening farms. While we are possibly not describing the full range of rearing systems, the farms included in the study should still cover the diversity and main approaches to the management of lambs and goat kids not intended for herd replacement on Swiss dairy farms.

### 4.1 Management aspects of the rearing system

Depending on the time the lambs and goat kids were kept with their dams, we grouped them into three rearing systems, namely, MB, TMB and ART. Systems using ART and MB rearing (with and without separation from the dam during certain times of day) have been described before (25, 41, 42) and are common rearing systems in Europe. In contrast, the only mention of TMB as rearing system outside of Switzerland (43) was described in the EFSA-report on welfare regarding livestock in Europe (44). In our study, around 25% of the lambs were reared TMB. Thus, TMB rearing seems to be a relevant system on Swiss small ruminant farms allowing prolonged contact of dam and young and the complete sale of the milk produced after separation of the young from its mother.

We found a large variety within these rearing systems regarding the duration of time lambs were kept with their mothers, milk feed provision (milk feed type, way of feeding, daily milk allowance, frequency of daily feeding), types of solid feed offered as well as age and weight at weaning and weaning strategies. Similar differences in artificially reared goat kids were reported in a survey conducted in 16 countries across three continents (28). With little research available on small ruminants in Central Europe, no definite best practice has been described in the literature for dairy farming of small ruminants. The situation of the dairy market, animal health issues or seasonal demands can lead to temporary changes in the rearing system and/or implementation of two or more rearing systems on the same farm in our study. Especially for goat kids, demand and prices for meat vary greatly between seasons and the carcass weight (45). Apparently, farmers of our study constantly evaluate the situation and adapt accordingly.

Another interesting aspect in our study was found in the fattening and marketing strategies of lambs compared to goat kids. Lambs from dairy sheep farms were frequently sold to fattening farms, and were commonly either slaughtered unweaned as suckling lambs or, more often, raised to a live weight of 40–50 kg. Their meat, which is in demand throughout the year (6), was mainly marketed via butcheries and bulk buyers. In contrast, goat kids were almost exclusively slaughtered at less than 12 weeks of age and often marketed directly, probably as a result of the lower, more seasonal demand for goat meat which peaks around Easter (6). Self-marketing makes farmers less dependent on the target goat kid slaughter weight of about 6.0 to 7.9 kg, which is enforced by bulk buyers with deviation being penalized (45). Unlike in countries such as France, where goat kids are commonly transported to specialized fattening farms within days of birth (24), this was the case only on one dairy goat farm in our study. However, this should be interpreted with caution. Dairy farms that fatten their lambs or goat kids on-site may be overrepresented because dairy farms relying in external fattening farms could not be included in this study if the associated fattening farm was not willing to participate.

### 4.2 Gamma globulin serum levels

We found lower gamma globulin serum levels in lambs (1.0 [0.77/1.14] g/dl) than in goat kids (1.3 [1.14/1.56] g/dl), which aligns with previous findings showing lower IgG in lambs (0.8 g/dL [(46)]) than in goat kids (1.2 g/dL [(17)]). The differences may be due to biological differences between the two species. While those previous studies measured IgG, the international standard for immune status, we measured gamma globulin due to financial constrictions. However, we consider the results comparable, as gamma globulin serum levels have been shown to closely correlate with IgG in equines (47, 48) as well as in lambs (49). Measurement of gamma globulin serum levels was shown to mostly produce slightly more conservative estimates as compared with IgG (47).

Adequate colostrum supply is crucial for both lambs (15) and goat kids (50) because immunoglobulin transfer occurs after birth (51). Thresholds of 1.5 g/dL of IgG for lambs and 1.2 g/dL of IgG for goat kids have been widely used to define failure of passive transfer (17, 42, 52, 53). The mean values of our results were above the threshold for goat kids and below the thresholds for lambs. However, most previous studies were conducted with meat breeds. Colostrum from dairy breeds tends to have. Lower IgG concentration (54). Thus, young from dairy breeds might generally have lower blood serum levels after the same amount of colostrum intake than those of meat breeds.

Gamma globulin serum levels varied widely in both species, ranging from 0.1 to 2.6 g/dL in lambs and 0.1 to 3.5 g/dL in goat kids and reflecting earlier findings [lambs: 0.1 to 3.8 g/dL (49), goat kids: 0.6 to 2.3 g/dL (55)]. This variance likely results from individual differences in colostrum quality (54), the amount ingested and absorbed by the lambs and goat kids (22) as well as a potentially the frequency of colostrum intake (56). No statistical effect of contact to the dam (MB vs. ART) at V1 was found in our study, likely because most farmers allowed their lambs or goat kids to suckle their dams for up to 5 days postpartum for ingestion of colostrum. Given the high variance in individual colostrum IgG, it may be advisable for farmers to assess the colostrum quality, for example by refractometry, and intervene if values are considered too low.

Gamma globulin serum levels in both lambs and goat kids were higher in younger animals and lower in older animals. This inverse relationship of gamma globulin serum levels and age is consistent with findings by Constant et al. (59), who observed a decline in IgG levels in goat kids during the first 3 weeks of life after an initial peak at 12 to 24 h. This decline occurs because ruminants do not produce their own immunoglobulins until later in life and can only absorb them from external sources during the first few days (15). Thus, the gamma globulin ingested from colostrum is gradually utilized and degraded over time (60).

We also expected lower gamma globulin serum levels in sick animals as has been found in other studies in lambs (19) and goat kids (18) and can be explained either by usage of gamma globulins during illness or increased susceptibility to illness due to low levels. However, we could not detect an effect of the health score at V1 on gamma globulin serum levels, which aligns with another study in which goat kids showed no correlation between health and immunoglobulin levels (22). Likely other factors such as good hygiene and low infection pressure may be able to mitigate any direct connection between gamma globulin serum levels and health. Another factor to consider is that potentially animals with very low health and gamma globulin died before our evaluation and a survival bias might be present. To fully evaluate the various influences, further research is required.

### 4.3 Health indicators

The overall average morbidity rates were 17.6% (V1), 30.5% (V2) and 22.2% (V3) in lambs and 11.5% (V1) and 16.5% (V2) in goat kids across the different visits. However, due to the nature of our study it was not feasible to assess if animals got sick between visits. In previous studies, a wide range of morbidity rates in lambs has been reported. Morbidity rates ranged from 1.6 to 86.6% in neonatal lambs (61), and a cumulative morbidity of 12.9% in lambs up to 3 months of age (62) and a preweaning morbidity of 27.3% in lambs (63) were found. Our results fall into that reported range. Our assessed morbidity rates in goat kids seem comparable or comparatively low to previous research [morbidity rates of 42.2% were described in goat kids from 0 to 30 days of age, dropping to 31.8% in goat kids aged 31 to 90 days and 26.0% in goat kids aged 91 to 180 days (64)]. The variance may be explained by the fact that some diseases such as orf or diarrhea can spread rapidly across the whole flock and thus affect many animals on a single farm while not affecting any animal on another farm.

The most common health issues in early life (V1) were umbilical disorders in lambs and fecal soiling or diarrhea in goat kids in our study. At V2, orf-like crusts and, in lambs, fecal soiling or diarrhea were most predominant. At V3, a reduction in respiratory quality was the main finding in lambs. Diarrhea and pneumonia were also reported by others as the main health issues of sick animals during the first 3 months of life (62, 65), whereas respiratory disease was found to increase with age and to be a significant cause of illness and death in feedlot lambs (23, 66). A good health score at V2 was positively related to ADG in lambs between V1 and V2, similar to the study by Lacasta et al. (23) where lambs with lung infections had a lower ADG than healthy lambs. Thus, promoting good health is important for farmers not just due to ethics and animal welfare but also out of economic considerations.

The overall mortality rate as calculated from the ATD was 11.9% in lambs and 6.1% in goat kids over the full course of our study. Previously found mortality rates ranged from 2% to up to 68.0% in lambs of various ages (12, 13, 61) and from 1.6% to up to 51% in goat kids (12, 14). Therefore, the mortality rates in lambs found in our study were in the expected range whereas mortality in goat kids was comparatively low. However, as opposed to our study, the previously mentioned studies included stillborn lambs and goat kids.

We found no statistical effects of the rearing system (in both lambs and goat kids), milk feed allowance, or place of fattening (in lambs only) on health scores at any of the visits, with the exception of the rearing system in goat kids at V2, where kids raised in the MB system showed better health scores than those in the ART system. Likely other factors such as infection pressure and general barn hygiene that were not considered in our analysis had an influence on the health outcomes. A potential influence of the place of fattening was expected because of stress and increased infection risk from transport and mixing of young animals from different farms (66–68). However, mortality was substantially higher in lambs transported to fattening farms before weaning (18.5%) than in those that remained on their birth farms (10.8%) or were transported to fattening farms after weaning (5.4%), suggesting a survival bias with sick or weak lambs possibly having died prior to health assessments, potentially masking any negative effects. However, this has to be interpreted with caution as 25% of the lambs sold to a fattening farm were fattened on only one fattening farm.

### 4.4 Average daily weight gain

The ADG in lambs during the first phase of rearing (V1–V2: 238 ± 86 g/day) was in the range of other studies with lambs until 30 days of age [223 to 240 g/day (69, 70)], whereas the ADG of lambs during the second phase of rearing (V2–V3: 196 ± 63 g/day) was low compared with another study (245 g/day) in which lambs were fattened from 2 months old until a slaughter weight of 18 kg at about 143 days of age (71). This relatively low average is likely due to the fact that some farms fattened their lambs extensively. We found no statistical effect of the rearing system during the first phase of rearing, whereas TMB lambs had a higher ADG than MB and ART lambs during the second phase of rearing. This effect is difficult to explain. Results from previous studies regarding the effect of the rearing system are controversial. Some found a higher ADG in ART than in MB lambs (69), whereas others found a higher ADG in MB than in ART lambs (72). Hernández-Castellano et al. (27) found the highest ADG in MB lambs until the start of the weaning period (at 10 kg), but differences in ADG disappeared across treatment groups during weaning. Apparently, aspects other than the rearing system itself such as breed, season, number of milk feeding bouts allowed or composition of the milk feed might possibly influence the ADG. For example, the composition of cow’s milk differs greatly from that of sheep’s milk (e.g., in energy value) and might therefore cause a reduced ADG when offered as milk feed in lambs (73).

Consistent with previous findings (74), a high milk allowance (ad libitum or temporarily ad libitum) toward the end of suckling had a positive effect on the ADG in lambs during the first phase of rearing but no effect during the second phase. Santos et al. (31), assessing lambs after weaning at 15 kg of weight, also found a higher ADG in ad libitum fed lambs than in those with restricted milk allowance.

Lambs that were transported to fattening farms before weaning had a lower ADG in the first rearing phase than those that remained on the dairy farms in our study. However, between V2 and V3, these lambs had an ADG comparable to that of lambs remaining on their dairy farms and much higher than that of lambs sold to fattening farms after weaning. The lower ADG in lambs that were transported to fattening farms before weaning is likely due to the previously discussed stress and increased infection risk from transport and mixing of young animals from different farms (66–68). Their high ADG in the second phase of rearing may again be due to the survival bias in lambs not transported to a fattening farm before weaning as discussed in the previous chapter. In comparison, the rather low ADG of the lambs being transferred to the fattening farms after weaning might then be the output of the more recent stress of the transfer. As Becker et al. (67) suggested in calves, certain management adjustments could help to alleviate the stress and health challenges faced on fattening farms, but the implications of this form of fattening and the optimal time of transfer to a fattening farm of these lambs should be investigated in more depth.

The ADG of 227 ± 62 g/day (range: 39 to 386 g/day) of the goat kids in our study was comparatively higher than in other studies [136 to 158 g/day (75, 76)]. Even though the ADG varied considerably between farms, we found no statistical effect of the rearing system, the milk feed allowance or the health status on the ADG. As in lambs, results from other studies are controversial. Some studies also found no differences in the ADG between goat kids in different rearing systems (75, 77), whereas others found a higher ADG in MB than in artificially fed goat kids (29). An explanation for our findings could be differences between breeds, quality of milk replacer or the fact that most of the goat kids in this study were fed with generous amounts of milk feed (ad libitum or temporarily ad libitum) toward the end of their suckling period, mitigating possible differences in milk feed amount between MB and artificially fed goat kids that might have been present in other studies.

### 4.5 Implications for welfare

To conclude on the rearing systems for lambs and goat kids on dairy farms, we must keep in mind that welfare encompasses not only good health and the absence of pain and suffering but also the ability to live reasonably natural lives and to experience positive affective states (78). In natural conditions, lambs and goat kids strongly bond to their mothers (16) and are typically suckled by their dams for 100 to 180 days until natural weaning (79). Lambs suckle their dams up to 36 times per day in the first few days of life and reduce the frequency to about 14 times per day at 6 to 7 weeks of age (80). This clearly differs from the situation the lambs and goat kids experienced on the dairy farms of our study, especially if lambs and goat kids were raised artificially. For example, if a restriction on milk feed amount or time was applied, the number of feeding bouts in our study was considerably low compared with natural conditions. The inability to behave naturally can lead to mutual suckling, and such behavioral disorder is considered a sign of impaired welfare (80). Although not further assessed, we observed mutual suckling on several occasions in ART lambs. Also, separation from the dam (81) or transport at a young age (80) can cause distress apparently without directly statistically affecting the ADG or health (but at least mortality in our study). Therefore, further studies with focus on behavioral indicators such as mutual suckling, playing time and vocalization (72, 81) are needed to comprehensively evaluate animal welfare in the different rearing and fattening systems in these animals.

## 5 Conclusion

This study investigated how different rearing systems and management practices on Swiss dairy farms affect gamma globulin serum levels, health, average daily weight gain (ADG) and mortality. While three rearing systems (MB, TMB, ART) were identified based on the length of dam contact, substantial variation in milk-feeding practices (e.g., feeding frequency, allowance, type) existed within these systems. Despite marked outcome variability, rearing system itself did not show a consistent effect on gamma globulin serum levels, health outcomes or ADG in either species. Gamma globulin serum levels were lower in lambs than in goat kids, probably due to species-specific differences, and were higher in younger animals than in older ones. Non-restrictive milk feeding, good health status at the second visit (V2), and absence of pre-weaning transport were associated with improved ADG of lambs during the first phase of rearing. In contrast, lambs transported to fattening farms before weaning showed higher ADG during the second phase of rearing, albeit with increased mortality compared to those that remained on their dairy farms. No rearing approach emerged as clearly superior across all indicators, suggesting that multiple management strategies may yield favorable results depending on context. However, unmeasured variables may also have contributed to outcome variability. As this study focused on performance-based indicators, which represent only one aspect of animal welfare, further research is needed to incorporate broader welfare indicators and assess long-term impacts. Future studies should aim to identify best practices that support both strong performance and positive welfare in dairy lambs and goat kids across diverse rearing systems.

## Data Availability

The raw data supporting the conclusions of this article will be made available by the authors, without undue reservation.
