# Supplementary material for: Management and performance of fattening lambs and goat kids in various rearing systems from Swiss dairy farms
Source: Front Vet Sci. 2025 Aug 4;12:1644500. doi: 10.3389/fvets.2025.1644500 (PMC12359181; doi:10.3389/fvets.2025.1644500)
Supplement: Supplementary file 1 [file Table_1.docx]

| Lambs | Initial sample | V1 | V2 | V3 | 365 days |
| --- | --- | --- | --- | --- | --- |
| Gamma globulin (n) | 543 | 131 | - | - | - |
| Health (n) |  | 539 | 476 | 379 | - |
| Average daily weight gain (n) |  | - | 476 | 379 | - |
| Slaughter age (n) |  | - | - | - | 396 |
| Mortality (n) |  | - | - | - | 521 |
| Mortality by place of fattening (n) |  | - | - | - | 508 |
| Goat kids | **Initial sample** | **V1** | **V2** | **V3** | **365 days** |
| Gamma globulin (n) | 247 | 86 | - | - | - |
| Health (n) |  | 235 | 212 | - | - |
| Average daily weight gain (n) |  | - | 212 | - | - |
| Slaughter age (n) |  | - | - | - | 194 |
| Mortality (n) |  | - | - | - | 229 |

Supplementary Material

Table S1: Initial sample size and sample sizes of lambs and goat kids at each visit for the assessment of gamma globulin serum level, health, average daily weight gain, and at 365 days of age for slaughter age and mortality.

Table S2: Minimum (Min), First Quartile (Q1), Median, Third Quartile (Q3) and Maximum (Max) values for gamma globulin serum level, health status for the three visits (V1, V2 and V3) and average daily weight gain (ADG) assessed for lambs and goat kids.

| Lambs | Unit | N | Min | Q1 | Median | Q3 | Max |
| --- | --- | --- | --- | --- | --- | --- | --- |
| Gamma globulin serum levels | g/dl | 131 | 0.1 | 0.5 | 0.9 | 1.3 | 2.6 |
| Health at V1 (farm level) | % | 539 | 0 | 8.3 | 15.8 | 23.7 | 43.3 |
| Health at V2  (farm level) | % | 476 | 0 | 12.5 | 30.8 | 38.4 | 83.3 |
| Health at V3  (farm level) | % | 379 | 0 | 8.8 | 20.0 | 31.0 | 53.8 |
| ADG for V1-V2 | g/day | 476 | 10 | 183 | 231 | 293 | 476 |
| ADG for V2-V3 | g/day | 379 | 29 | 153 | 195 | 231 | 377 |
| ADG for V1-V3 | g/day | 379 | 87 | 179 | 206 | 240 | 349 |
| Goat kids | **Unit** | **N** | **Min** | **Q1** | **Median** | **Q3** | **Max** |
| Gamma globulin serum levels | g/dl | 86 | 0.1 | 0.8 | 1.3 | 1.8 | 3.5 |
| Health at V1 (farm level) | % | 235 | 0 | 5.3 | 8.3 | 11.1 | 75.0 |
| Health at V2  (farm level) | % | 200 | 0 | 0 | 10.8 | 15.7 | 100.0 |
| ADG for V1-V2 | g/day | 200 | 39 | 189 | 221 | 264 | 386 |
